# Which Presentation Speed Is Better for Learning Basketball Tactical Actions Through Video Modeling Examples? The Influence of Content Complexity

**DOI:** 10.3389/fpsyg.2019.02356

**Published:** 2019-10-22

**Authors:** Mohamed Jarraya, Ghazi Rekik, Yosra Belkhir, Hamdi Chtourou, Pantelis T. Nikolaidis, Thomas Rosemann, Beat Knechtle

**Affiliations:** ^1^UR15JS01, EM2S, Education, Motricité, Sport et Santé, High Institute of Sport and Physical Education, University of Sfax, Sfax, Tunisia; ^2^Institut Supérieur du Sport et de l’Education Physique de Sfax, Université de Sfax, Sfax, Tunisia; ^3^Activité Physique, Sport et Santé, UR18JS01, Observatoire National du Sport, Tunis, Tunisia; ^4^Exercise Physiology Laboratory, Nikaia, Greece; ^5^Institute of Primary Care, University of Zurich, Zurich, Switzerland; ^6^Medbase St. Gallen Am Vadianplatz, St. Gallen, Switzerland

**Keywords:** videos, instructional designs, human movement, tactical learning, physical education

## Abstract

The present experiment examined the effect of content complexity on perceived cognitive load and game performance when learning basketball tactical actions from videos modeling examples displayed at different speeds. A two (presentation speed: slow vs. normal) × three (content complexity: low vs. medium vs. high) design between subjects was adopted in the experiment. Following the learning phase, 120 secondary school students were quasi-randomly assigned to six experimental conditions and required to rate their perceived cognitive and game performance. Data analyses revealed that for low complexity content, both speeds of presentation have similar effects on learning. Conversely, for medium and high complexity contents, participants exposed to the slow-presentation speed learned more efficiently than those exposed to the normal-presentation speed. The findings recommend the use of slow-speed videos when learning basketball tactical actions, particularly in playing systems with medium or high levels of complexity.

## Introduction

Learning playing systems in basketball depends heavily on the students’ ability to construct a coherent mental representation that exactly depicts tactical actions (e.g., screening, acceleration, movements, etc.). With the increasing use of video modeling examples during physical education (PE) lessons (e.g., Zetou et al., 2002; Barzouka et al., 2015), teachers can rely on such dynamic visualizations in order to explain, as precisely as possible, the tactical actions depicted in diverse playing systems. In spite of the exponential use of video modeling examples in PE domain, little is still known about how teachers should design them in order to improve tactical learning of students. The overarching research question of this study was: which presentation speed is better in helping students to learn basketball tactical actions through video modeling examples?

Video modeling examples are believed to have an enormous potential for sustaining students’ learning, because they can deliver the information concerning how to perform skills perfectly (Hoogerheide et al., 2016). Research in PE setting has validated the value of these dynamic visualizations in improving motor skills. For example, evidence of positive effects of video modeling examples was obtained for the acquisition and retention of the set and serve skills in volleyball (Zetou et al., 2002). Similarly, viewing a skilled model was more effective than oral explanations when learning about the skill of setting in volleyball (Vernadakis et al., 2006). Moreover, model-based video has been observed to be better than simple verbal commentaries to learn the shooting skill in handball (Nahid et al., 2013). More recently, Barzouka et al. (2015) proved that observing a skilled model performing the skill of passing in volleyball was more beneficial than oral explanations. According to the neuroscience literature, positive effects of video modeling examples on learning motor skills are mainly due to the activation of the Mirror-Neuron System (MNS). This system is a neurophysiological circuit distributed across the pre-motor cortex that is automatically activated when someone is observing another person performing an action (see Buccino et al., 2004; Rizzolatti and Craighero, 2004; Turella et al., 2009; Van Gog et al., 2009).

However, despite these claims, recent evidence in the education field has failed to confirm the effectiveness of video modeling examples in learning diverse motor skills such as making origami shapes (Wong et al., 2012), constructing 3D Lego figures (Wong et al., 2015), and executing sutures procedure (Ganier and De Vries, 2016). According to these researchers, instructional videos involving human movements can become subject to transience effects (Wong et al., 2012). The transient information effect can be observed with dynamic visualizations that provide a non-permanent flow of information that disappears from the computer screen. The transient nature of information requires that learners have to process new information while simultaneously integrating previously presented information (see Ayres and Paas, 2007a, b; Sweller et al., 2011). This mental process imposes high extraneous cognitive load on limited resources of working memory (WM) (Sweller et al., 1998, 2011; Lowe, 1999; Hegarty, 2004). As it is well known, the WM is very limited in both capacity and duration when processing new information (Baddeley, 2003). Therefore, it seems very tricky to process simultaneously more than a few novel elements of information without a potential WM overload (Cowan, 2001). As a result, learners may miss important information required to build a coherent mental model of the diffused content.

In order to avoid the transient information effect and improve learning from dynamic visualizations, research using the cognitive-load theory (CLT) has proposed several design guidelines (for a review, see Khacharem et al., 2015). One of these design guidelines is *“decreasing the presentation speed”* which recommends displaying videos or animations at a slow speed rather than at a normal speed. Despite the limited number of studies, the positive effect of such kind of temporal manipulation is well established. For example, in a learning environment about the behavior of a *five-ball Newton’s Cradle*, Lowe (2006) proved that participants in the slow-speed group give more explanatory accounts or made inferences more than participants in the normal-speed group. Moreover, Meyer et al. (2010) investigated the effects of user-controlled presentation speed when learning about the functioning of a four-stroke engine. Findings demonstrated that students preferred slow-speed animation in order to observe micro-level events of the animation. In another study conducted in the sports and coaching domain, novice footballers achieved higher recall scores, needed a lower number of repetitions and had to invest less mental effort when the animations were played at a slow speed compared to a normal speed (Khacharem et al., 2014). According to these studies, displaying animations with a slow speed provide learners with more time to encode the diffused information into WM. This mental process reduces the probability that relevant information is missed or ignored (Lowe, 2006). Furthermore, decreasing the animation’s presentation speed may be beneficial as it reduces the perceptual and cognitive demands by allowing learners to build a mental representation of local parts, which then can be integrated into a coherent mental model (Meyer et al., 2010). In other words, animations played at a slow speed alters the perceptibility profile of the communicated information, so that the changes that occur at the micro level of the dynamic phenomenon become more perceivable, which may promote better understanding (Khacharem et al., 2014). Additionally, the instructional benefits of decreasing the animated presentation speed were supported by Moriuchi et al. (2014) when observing motor skills (manipulative task). In this investigation, authors proved that the MNS or the action observation system (as termed by the authors) became more active when the subject observed the video clip at a slow speed rather than at a normal speed.

According to Bissonnette and Richard (2005), a regular process of learning is composed by three main phases: acquisition, retention, and a transfer phase where teachers are asked to increase the cognitive complexity of the previous proposed tasks/situations (i.e., from simple to complex context). For instance, in tactical scenes of play, adding the number of players as well as the number of interactions between them (i.e., their relative movements) is a useful means of increasing the cognitive complexity of a situation (Raab, 2002, 2003).

With the increasing use of videos modeling examples in PE lessons to depict explicitly dynamic behaviors such as motion, jump, and throw, these external visualizations look well suited to teaching or revising complex tactical scenes of play that are often difficult to describe verbally by PE teachers. However, dealing with complex videos could be a very challenging task for novice learners due to their limited WM resources resulting in significant cognitive demands. Following the expert-novice paradigm, it is well known that experts have better cognitive capacity than novices making them more efficient in the identification/recollection of relevant information, and enabling them to make quicker and more appropriate decisions (e.g., Rink et al., 1996; Dodds et al., 2001; Nielsen and McPherson, 2001; Moran, 2013).

The question that arises here is how to present video modeling examples in order to promote optimal memorization of various playing systems in novice learners?

The present study addresses this issue by examining the influence of content complexity on learning various tactical actions in basketball through videos displayed at slow and normal speeds.

This experiment was novel in two ways. Firstly, no previous research has examined the role of the presentation speed when learning technical and/or tactical skills from videos modeling examples in PE context. Secondly, the available evidence (see Moriuchi et al., 2014) about the effect of these modes of videos’ presentation speed when observing motor skills is focused only on a manipulative task (i.e., catching a ball). Moreover, in this study, authors have not taken into account either the complexity of the displayed task or the learning of the motor skill.

Based on the literature reviewed in the previous section, it was hypothesized that whatever the content complexity the videos with a slow presentation speed would lead to more efficient learning (i.e., higher game performance scores with lower investment of mental effort) compared to a normal presentation speed.

## Materials and Methods

### Participants and Design

There were 120 secondary school students that volunteered to participate in this study. There were 78 females and 42 males, with an average age of 15.42 years (*SD* = 0.49). The participants were recruited (in collaboration with eight PE teachers) based on the following inclusion criteria: (i) No previous experience in playing basketball or any other team ball sports in a club—this requirement was applied to ensure that all students were domain novices and to keep away the effects of transfer across sports (Smeeton et al., 2004)—and (ii) having normal or corrected to normal levels of visual function. The study protocol was approved by the local Institutional Ethics Committee, and participants were informed that they could leave the experiment at any time while preserving their anonymity.

A two (presentation speed: slow vs. normal) × three (content complexity: low vs. medium vs. high) design between subjects was adopted in the experiment. The participants were quasi-randomly allocated to one of six experimental conditions, so that each group contained equal numbers of males (7) and females (13).

### Apparatus

The apparatus consisted of an ASUS laptop (X507UA) placed at a distance of 30 cm from the participant. The stimulus was presented on a 34 × 22 cm screen, with approximately 45° viewing angle.

### Instructional Material

**FIGURE 1:**
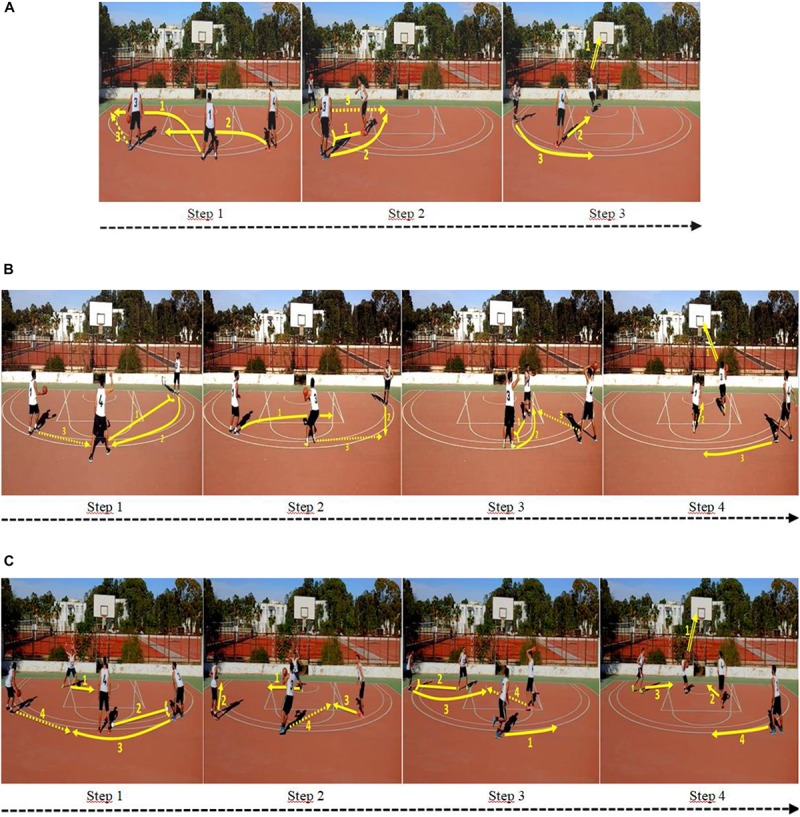
Screenshots showing the key steps of the basketball playing systems: **(A)** simple, **(B)** moderate, **(C)** complex (arrow symbols were incorporated in screenshots just to indicate the actions of play: dashed arrow = simple pass; solid arrow = movement; double solid arrow = layup; solid arrow with short perpendicular line = movement for screen).

Participants in the experiment were required to learn various tactical actions (e.g., screening, movements, and layup) depicted in three offensive playing systems in basketball with different levels of complexity. The term “*complexity*” adopted in this experiment refers to the internal complexity of the situation, that is, the intrinsic cognitive load associated with it (e.g., Pollock et al., 2002). To manipulate the complexity of the basketball game systems, we followed the recommendations of cognitive load theory, by varying the amount and the connectivity of the presented information (on this point, see Sweller et al., 1998). The three playing systems were designed firstly in collaboration with an experienced basketball coach. Then, another two experienced basketball coaches independently rated on a 5-point Likert scale each sequence in relation to whether the pattern of play corresponded to a structured zone attack scene (0 = *very unstructured*; 5 = *very structured*). All sequences were rated 4 or above and thus were kept for the experimentation. Finally, these playing systems were rated according to their complexity by these two experienced basketball coaches. Note that all coaches were, at the same time, PE teachers and coaches licensed by the Tunisian Basketball Federation and had satisfactory experience at a professional level (*M* = 15 years, *SD* = 1.67) (see Figure 1).

• The playing system with low complexity included three players—a playmaker➀, a pivot➃, and a winger➂—who carried out a simple zone attack, composed of two passes before a basket was taken through a layup.
• The playing system with medium complexity included three players—a playmaker➀, a pivot➃, and a winger➂—who carried out a moderate zone attack, composed of three passes before a basket was taken through a layup.
• The complex playing system with high complexity included four players—a playmaker➀, two pivots➃/➄, and a winger➂—who carried out a complex zone attack, composed of four passes before a basket was taken through a layup.

To test our hypotheses, two versions were developed for each playing system. The first version showed the evolution of the three zone-attacks at normal speed (1.0) (based on the standard speed of the camera). To develop this version, professional players (*M*_age_ = 21.7 years, *SD* = 1.26), serving as models, was filmed from a camera placed at the middle of the field in an elevated position (3 m). This camera position made it possible to view the entire field of play as well as the players. The durations of the simple, moderate and complex playing systems were 8, 12, and 16 s, respectively, before vanishing from the screen.

The second version showed the evolution of the three zone-attacks at low speed. Here, the developed videos (i.e., with normal speed) were edited within *Adobe Premiere Elements 9*, to slow them down to 0.5 times normal speed. The durations of the simple, moderate and complex playing systems were 16, 24, and 32 s, respectively, before vanishing from the screen.

To avoid the occurrence of modality, redundancy or temporal continuity effects, the instructional materials were purely visual (i.e., contained no spoken commentary or sound) and system paced (Kalyuga, 2008).

### Procedure

The experiment was carried out in groups of 18 to 19 participants during regular PE sessions (no more than 60 min). In each group, students were tested individually with the experimenter observing. Before beginning experimental sessions, students were given a brief account of what was required in the study and ethics protocols completed. Next, they completed a questionnaire that solicited demographic information and asked about their previous experience with basketball/other team sports, and video games related to team ball sports. After that, each participant was quasi-randomly allocated to one of six experimental conditions and was instructed to memorize as precisely as possible the evolution of the scene of play (shown twice). Immediately following this learning phase, student was given 2 min to self-report the mental effort investment level associated with the learning task and to perform the game comprehension test. The experimenter timed this using a hand-held stopwatch.

### Dependent Measures

This study incorporated three dependent variables, including mental effort (1–9), game performance (score) and learning efficiency (score).

### Mental Effort

The reliable and valid mental effort scale of Paas (1992) was used to obtain an indication of cognitive load investment. On a sheet of paper, students were required to tick a box on 9-point scale ranging from (1) “*very low*” to (9) “*very high.*” The statement was “*please indicate how much mental effort you invested to learn the scene of play.*”

### Game Performance

Students were invited to use the knowledge obtained from the instructional material and apply it on a basketball half-court. Here, participants were instructed to reproduce as accurately as possible the actions performed by a randomly chosen player from the instructional material (i.e., playmaker, pivot, or winger). The test was conducted with other male semi-professional basketball players aged between 15 and 16 years old who already knew the progress of all the playing systems. To guarantee a smooth running of the task, one of the players was selected to intervene by providing a verbal corrective feedback each time the student performed a wrong action. The students’ actions were filmed from a digital camera (position 1 m above the ground from the middle of the field). Then, two independent raters scored the total number of correct and incorrect position/action that each student performs in the field. The inter-rater reliability analysis showed almost perfect agreement. Cohen’s (κ) values across low, medium, and high complexity contents were as follows: 0.92, 0.89, and 0.9. For each correct position/action in the game performance test, the students were assigned one point with a maximum score of six points for a simple playing system, and eight points for moderate/complex playing systems, otherwise they received zero points.

### Learning Efficiency

Mental effort scores were combined with game performance scores to calculate instructional efficiency based on the computational approach developed by Kalyuga and Sweller’s (2005). According to this approach, a lower rating of mental effort combined with higher game performance scores would provide evidence of a more efficient learning condition.

Learning efficiency=game performance/mental effort

### Data Analysis

Data were analyzed using the Statistica software (*StatSoft, France; version 10*). After verifying the normality of the distribution using Shapiro–Wilk test, a two-way design analysis of variance (ANOVA) has been applied, with presentation speed (slow vs. normal) and content complexity (low vs. medium vs. high) as between-subject factors. Effect sizes were expressed as partial eta squared (${\text{η}}_{p}^{2}$) to assess the potential practical significance of the findings. The Bonferroni *post hoc* test was utilized for the comparison of the data. Effect size (Cohen’s *d*) and the coefficient of variation (CV%) for pair wise comparisons were also calculated. Partial eta squared values of 0.01, 0.06, and 0.14 and Cohen’s *d* values of 0.20, 0.50, and 0.80 represent small, moderate, and large effect sizes, respectively. The alpha level for significance was set at *p* < 0.05 for all analysis, and data are presented as means (SD).

## Results

Descriptive statistics for effort mental, game performance and learning efficiency, for each experimental condition are presented in Table 1.

**TABLE 1:** Mean ± SD scores and the coefficient of variation (CV%) concerning mental effort (ME), game performance (GP), and learning efficiency (LE) as a function of the presentation speed and the content complexity.

	**Slow-presentation speed**			**Normal-presentation speed**		
	**Low**	**Medium**	**High**	**Low**	**Medium**	**High**
ME	5.05 ± 0.6 (CV% = 11.9)	5.35 ± 0.67 (CV% = 12.5)	5.8 ± 0.89 (CV% = 15.4)	5.3 ± 0.66 (CV% = 12.4)	6.5 ± 0.95 (CV% = 14.5)	6.9 ± 0.97 (CV% = 14.0)
GP	5.5 ± 0.61 (CV% = 11.0)	6 ± 0.92 (CV% = 15.3)	5.75 ± 0.72 (CV% = 12.4)	5.35 ± 0.49 (CV% = 9.1)	4.9 ± 0.79 (CV% = 16.1)	4.6 ± 0.6 (CV% = 13.0)
LE	1.1 ± 0.16 (CV% = 14.7)	1.13 ± 0.17 (CV% = 14.8)	1.01 ± 0.19 (CV% = 18.8)	1.02 ± 0.15 (CV% = 14.4)	0.77 ± 0.16 (CV% = 21.2)	0.68 ± 0.12 (CV% = 18.2)

### Mental Effort

Analysis of variance revealed a significant effect of presentation speed [*F*(1.19) = 46.57, *p* < 0.001, ${\text{η}}_{p}^{2}$ = 0.71] a significant effect of content complexity [*F*(2.38) = 23.37, *p* < 0.001, ${\text{η}}_{p}^{2}$ = 0.55] and a significant interaction between these two factors [*F*(2.38) = 3.80, *p* < 0.05, ${\text{η}}_{p}^{2}$ = 0.09]. Further analysis indicated that when the content complexity was low, students invest the same mental effort whatever the levels of presentation speed (*p* > 0.05). However, for contents with medium and high complexity, students invest less mental effort with a slow presentation speed than with a normal presentation speed (medium complexity: *p* < 0.01, *d* = 1.4; high complexity: *p* < 0.001, *d* = 1.2).

### Game Performance

Analysis of variance revealed a significant effect of presentation speed [*F*(1.19) = 27.77, *p* < 0.001, ${\text{η}}_{p}^{2}$ = 0.59], a non-significant effect of content complexity [*F*(2.38) = 1.90, *p* < 0.01, ${\text{η}}_{p}^{2}$ = 0.18] and a significant interaction between these two factors [*F*(2.38) = 7.87, *p* < 0.01, ${\text{η}}_{p}^{2}$ = 0.29]. Further analysis indicated that when the content complexity was low, students achieved equal game performance scores whatever the levels of presentation speed (*p* > 0.05). However, for contents with medium and high complexity, students achieved higher game performance scores with a low presentation speed than with a normal presentation speed (medium complexity: *p* < 0.001, *d* = 1.3; high complexity: *p* < 0.001, *d* = 1.7).

### Learning Efficiency

**FIGURE 2:**
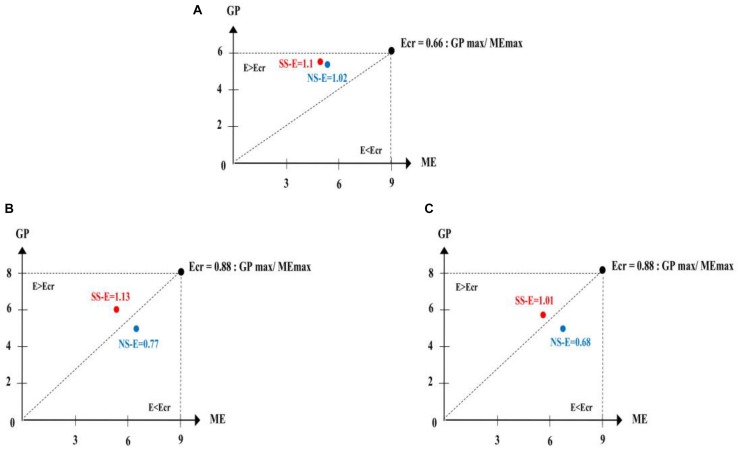
Learning efficiency as a function of presentation speed and content complexity (**A** = low complexity, **B** = medium complexity, **C** = high complexity). The diagram is a representation after Kalyuga and Sweller (2005), where ME = mental effort, GP = game performance, SS-E = efficiency with a slow-speed, NS-E = efficiency with a normal-speed, and Ecr = critical efficiency.

Analysis of variance revealed a significant effect of presentation speed [*F*(1.19) = 75.58, *p* < 0.001, ${\text{η}}_{p}^{2}$ = 0.5], a significant effect of content complexity [*F*(2.38) = 15.11, *p* < 0.001, ${\text{η}}_{p}^{2}$ = 0.44] and a significant interaction between these two factors [*F*(2.38) = 10.22, *p* < 0.001, ${\text{η}}_{p}^{2}$ = 0.35]. Further analysis revealed no significant differences between the low and the normal levels of presentation speed (*p* > 0.05) when the content complexity was low. Conversely, for the contents with medium and high complexity, learning was more efficient with the low presentation speed than with the normal presentation speed (medium complexity: *p* < 0.001, *d* = 2.2; high complexity: *p* < 0.001, *d* = 2.1). Representations after Kalyuga and Sweller’s (2005) of learning efficiency as a function of presentation speed and content complexity are illustrated in Figure 2.

## Discussion, Limitations and Future Directions

The present study tested the effect of two presentation speed as a function of content complexity on learning of basketball tactical actions through video modeling examples. To explore relationships between these two factors, a game performance test based on the recall reconstruction-paradigm of Chase and Simon (1973) and the mental effort 9-point scale of Paas (1992) were used. It was predicted that whatever the content complexity, the students profited more from the slow than the normal presentation speed. This prediction was partially confirmed.

On the one hand, decreasing the video presentation speed has no benefits for learning the content with low complexity: students achieved the same level of game performance with the same investment of mental effort. These results could be interpreted from the CLT viewpoint (Sweller et al., 1998). Indeed, dynamic visualizations (videos or animations) that diffused simple contents leads to easier learning, because learners have to consume less perceptual-cognitive resources to deal with both the transient nature of information and the few numbers of interactive elements (i.e., players and their actions). As a result, learners were not forced to integrate and maintain several information elements in WM, and may effectively benefit from videos played at a normal speed without missing any decisive information.

On the other hand, results demonstrated that decreasing the video presentation speed is an effective design technique when learning contents with medium and high complexity: students obtained higher game performance scores with lower investment of mental effort when the video was played at a slow speed than when it was played at a normal speed. According to CLT, dealing with dynamic visualizations having a certain degree of complexity could be a very tricky task for novice learners due to their limited WM resources. Challenges arise from the excessive number of interactive elements and from the transient nature of information, that requires students to temporarily hold the video frames in WM to mentally link them (Lowe, 2003; Sweller et al., 2011). The advantage of displaying videos at slow speed is that it may reduce these cognitive processing demands by providing learners with additional time to process and integrate the diffused content, which may in turn lead to reduce the probability that essential information was missed (Khacharem et al., 2015). Furthermore, displaying videos at a slow speed may have highlighted micro-events by making the detailed information that occurs within the basketball playing-systems more perceivable (Lowe, 2006; Meyer et al., 2010). In other words, the evolution of the scene of play from one step to another consisted of a series of micro-events (e.g., screening, acceleration, movements, and layup). It is probable that these micro-events could be correctly perceived, processed and integrated in long-term memory structures due to the decrease of the presentation speed (Khacharem et al., 2014).

Overall, the current study reported mixed effects for the use of videos with different levels of speed when taking the factor of content complexity into account. These findings are interesting in that they could be explained by the results reported by Moriuchi et al. (2014). Indeed, authors proved that the mirror neuron system became more active when observing a video clip involving motor skills at a slow speed rather than at a normal speed. Although the authors did not consider the level of content complexity, we could predict that the used instructional material was sufficiently complex for learners. Taking into account the mixed results founded in the present study, further researches are needed to measure the activation of MNS while considering the characteristics (i.e., content complexity) of the displayed tasks.

Despite the important results reported in the present investigation, certain possible limitations of this study should be kept in mind when interpreting the results. Firstly, the present study does not take into consideration learners’ individual differences, particularly gender. It has been established that females benefited more than males from using instructional videos involving motor skills (see Wong et al., 2015). Future studies need to investigate the potential interplay between the gender of learners and the presentation speed when learning motor skills through video modeling examples. Secondly, the game performance test was applied immediately after the learning phase. It would be worthwhile in future studies to look at how this pattern of results remain valid or not after an interfering test such as paper folding test (see Ekstrom et al., 1976) in order to measure long-term learning. Lastly, it has been established that dynamic visualizations (e.g., video modeling or video feedback) could be successfully used during PE lessons as mediums to enhance students’ attitudes such as enjoyment, engagement and motivation (e.g., Casey and Jones, 2011; Potdevin et al., 2018; Rekik et al., 2019). It would be interesting in further research to explore if students’ attitudes change as a function of the video’s presentation speed when learning basketball playing systems with different levels of complexity.

## Conclusion

In summation, the present research offers insight into the role of decreasing the presentation speed when learning basketball tactical actions from video modeling examples. The results encourage PE teachers to use slow-speed videos, particularly when presenting basketball playing systems with medium and high complexity.

## Data Availability Statement

The datasets generated for this study are available on request to the corresponding author.

## Ethics Statement

The studies involving human participants were reviewed and approved by the local Institutional Ethics Committee, and participants were informed that they could leave the experiment at any time while preserving their anonymity. Written informed consent was obtained from the minor(s)’ legal guardian/next of kin for the publication of any potentially identifiable images or data included in this article.

## Author Contributions

MJ and GR conceived the study. MJ, GR, YB, HC, PN, and BK designed the study. GR and YB collected the data. MJ, GR, and YB analyzed and interpreted the data, and drafted the manuscript. HC, PN, TR, and BK revised the manuscript and approved the final version.

## Conflict of Interest

The authors declare that the research was conducted in the absence of any commercial or financial relationships that could be construed as a potential conflict of interest.
